# Structure and regulation of MARK, a kinase involved in abnormal phosphorylation of Tau protein

**DOI:** 10.1186/1471-2202-9-S2-S9

**Published:** 2008-12-03

**Authors:** Thomas Timm, Alexander Marx, Saravanan Panneerselvam, Eckhard Mandelkow, Eva-Maria Mandelkow

**Affiliations:** 1 Max-Planck-Unit for Structural Molecular Biology, c/o DESY, Notkestrasse 85, 22607 Hamburg, Germany

## Abstract

Protein kinases of the MARK family phosphorylate tau protein in its repeat domain and thereby regulate its affinity for microtubules and affect the aggregation of tau into Alzheimer paired helical filaments. We are searching for low molecular weight compounds to interfere with the activity of MARK and its pathways. Here we summarize structural features of MARK and cellular pathways of regulation.

## Introduction

Kinases of the MARK (microtubule-associated protein (MAP)-microtubule affinity regulating kinase)/Par-1 family play important roles in several contexts relevant for Alzheimer's disease (AD) research. The best-known property is that these kinases phosphorylate the tau protein in its repeat domain. The consequence is that tau looses its affinity for microtubules and detaches from them. The microtubules become destabilized, and the unbound tau is free to undergo abnormal aggregation. This process represents one of the hallmarks of AD. Furthermore, the sites on tau phosphorylated by MARK (KXGS motifs in the repeat domain) appear early in AD [1], MARK protein is elevated in neurofibrillary tangles in AD brain [2], and MARK phosphorylation sites on tau are elevated early in transgenic mouse models of tauopathy [3,4].

Apart from neurodegeneration, MARK and its homologues belong to a set of conserved proteins that are essential for establishing cellular polarity, which is crucial for the development of an organism. Because of this property, these proteins were initially named Par (for 'partition-defective'), since mutations in the gene products lead to defects in the partitioning of the *Caenorhabditis elegans *zygote [5]. The combination of Par genes has since been discovered and studied in many contexts, notably in polarity development in the fruit fly [6] and in the establishment of polarized epithelial cells [7]. Besides tau, a number of other MARK target proteins have been identified. They include the MAPs related to tau (for example, MAP2, MAP4 and their isoforms) [8], other MAPs (for example, doublecortin), or proteins involved in phosphorylation signaling and 14-3-3 binding (for example, Cdc25, PTPH1, and KSR1) [9]. In particular, the Par genes are involved in neuronal differentiation [10], and the activity of MARK2/Par-1 is necessary for the outgrowth of cell processes, neurites, and dendritic spines [11-13].

Our search for MARK was prompted by the observation of the phoshorylated KXGS motifs in tau protein and their strong effect on microtubule affinity. The kinase was identified and cloned on the basis of this property [14,15]. The kinase subfamily contains four members, termed MARK1–4, encoded on chromosomes 1, 11, 14, and 19 in the human genome. MARK belongs to the AMPK (adenosine-monophosphate activated protein kinase) branch of the CAMK (calcium/calmodulin-dependent protein kinase) group of kinases [16]. The kinase is relatively large (nearly 800 residues), contains several domains, and appears to be regulated by multiple pathways. Two of these were already anticipated when the kinase was originally isolated because two residues in the so-called 'activation loop' of the catalytic domain were phosphorylated. Subsequent work showed that phosphorylation of the first site (T208 in the MARK2 sequence) activated the kinase, whereas the second site was inhibitory (S212). Activation by phosphorylation at T208 can be achieved by the upstream kinase MARKK/TAO-1 [17], or alternatively by the kinase LKB1 [18]. Inactivation by phosphorylation at S212 is achieved by the glycogene synthase kinase 3β (GSK3β) [19]. Because of the relationship to tau phosphorylation in neurofibrillary pathology, we are interested in low molecular weight compounds by which the activity of MARK can be modulated. One example is that of hymenialdisine, which inhibits MARK and other kinases by binding in the catalytic pocket [12,20]. Here we summarize some structural features of MARK and mechanisms of regulation that may serve as entry points for pharmacological intervention.

## MARK structure

**Figure 1:**
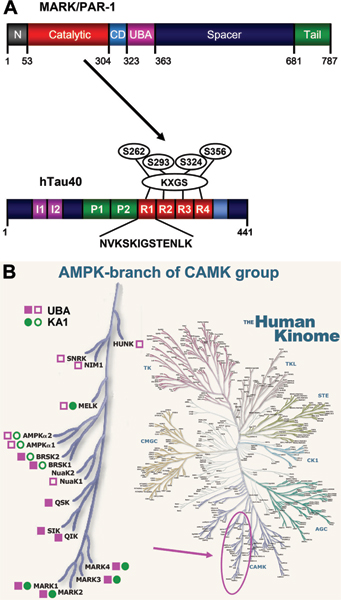
Diagrams of MARK and tau. **(A) **Top: bar diagram representing the domain structure of MARK family kinases. Residue numbers refer to the sequence of the longest isoform of human MARK2. The domains include the header domain, the kinase catalytic domain, the common docking (CD) site, the ubiquitin-associated (UBA) domain, the spacer domain, and the tail domain, which contains the 'kinase-associated domain' (KA1). Bottom: bar diagram of tau protein, highlighting the repeat domain and the KXGS motifs that can be phosphorylated by MARK. **(B) **Phylogenetic branch of the AMPK-related kinases and their position in the human kinome (modified from [16]). Closed squares and circles indicate UBA and KA1 domains recognized on the basis of sequence similarity by Prosite [36]. Open squares and circles label kinases where UBA and KA1 domains are not predicted by sequence, because the homology is too weak, but only on the basis of secondary structure elements (determined using PSIPRED [37]).

The major domains of MARK (Figure 1A) include an amino-terminal header, the kinase catalytic domain, the ubiquitin-associated (UBA) domain, a spacer domain, and the carboxy-terminal tail domain (containing the 'kinase-associated domain' (KA1)). All four MARK isoforms have a similar domain composition. UBA domains can be found by sequence homology in several other AMPK-related kinases, such as the salt-induced kinases SIK1–3 (also termed SIK, QIK and QSK) and the brain-selective kinases BRSK1/2 (also termed SAD kinase) [21]. Predictions based on structure suggest 3-helix bundles resembling the UBA domain at analogous positions for several further kinases of the AMPK family, such as NUAK1/ARK5 (AMPK-related kinase 5; but not NUAK2/SNARK (sucrose non-fermenting 1 (SNF1)/AMPK-related kinase)), AMPKα1/2, MELK (maternal embryonic leucine zipper kinase), NIM1 (non-inducible immunity 1), SNRK (SNF1-related kinase), and HUNK (hormonally upregulated Neu-associated kinase) (Figure 1B).

The carboxy-terminal KA1 domains are only predicted for a subset of the AMPK family kinases, MARK1–4, AMPKα1/2, BRSK1/2 (alias SADK-A/B) and MELK (Figure 1B). Among the domains of MARK, three have been solved structurally at high resolution: the catalytic domain together with the UBA domain (by X-ray diffraction) [22-24]; and the tail domain (by NMR spectroscopy) [25]. If not stated otherwise, residue numbers refer to MARK2 [UniProt: Q7KZI7], which is the isoform that has been resolved at the highest resolution so far [PDB: 2Y8G].

**Figure 2:**
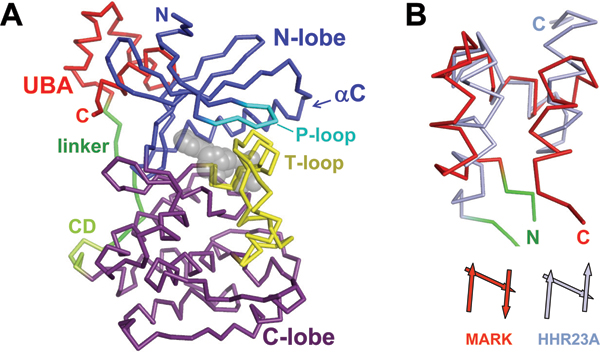
Structure of the catalytic and ubiquitin-associated (UBA) domain of MARK. **(A) **Cα trace of the inactive catalytic domain of MARK with carboxy-terminal extension, in the inactive apo state. The nucleotide binding site is marked by an ATP molecule represented by transparent spheres (grey). The catalytic domain is drawn in blue (amino-terminal lobe) and purple (carboxy-terminal lobe) except for the P-loop (cyan) and the T-loop or activation loop (including its amino-terminal anchor; yellow). The drawing is based on the coordinates of MARK1 [PDB: 2HAK] [23]. The activation segment is variable and, in most cases, partially invisible in the crystal structures. Two complete conformations of the T-loop are shown in superposition; these correspond to molecules E and F of the MARK1 crystal structure. The carboxy-terminal extension of the catalytic domain comprises the common docking (CD) site and linker (pale green and green) and the UBA domain (red), which binds to the amino-terminal-lobe, at the rear side of the active site. **(B) **Top: Overlay of the UBA domain of MARK1 (red) and that of HHR23A (light blue) [PDB: 1IFY] [38]. Bottom: schematic drawing showing the orientation of the helices in both structures. The carboxy-terminal helix (alpha3) of the UBA domain in MARK is inverted compared to the conventional orientation exemplified by HHR23A. The figure was generated with PyMOL [39].

The catalytic domain (Figure 2A) has a bi-lobal structure typical of protein kinases, with a conserved active site cleft between the two lobes. The smaller amino-terminal lobe (approximately 80 residues) consists mainly of β-strands (β1 to β5) and a single, prominent α-helix (helix C), and the larger carboxy-terminal lobe (approximately 170 residues) is mostly α-helical. Both lobes contribute structural elements to the active site that are essential for the catalytic activity: the P-loop (phosphate-binding loop, also known as glycine-rich loop, G-loop), the catalytic loop, and the activation loop (also called T-loop). The P-loop, at the tip of the β1–β2 hairpin (GKGNFA in all MARK isoforms, residues 60–65 in MARK2), is rather flexible in the apo-state. In the active complex it locks in to the nucleotide and helps to position the γ-phosphate of the nucleotide for transfer to the substrate's phosphorylation site (by analogy to known structures of active protein kinase complexes). The catalytic loop (residues 171–183), part of the C-lobe, is located at the other side of the catalytic cleft, opposite to helix C and the P-loop. The catalytic loop contains an RD motif consisting of a highly conserved, catalytically active aspartate (D175), which is preceded by an arginine residue. In RD kinases that are regulated by T-loop phosphorylation, this arginine is assumed to interact with the primary phosphorylation site of the T-loop [26].

**Figure 3:**
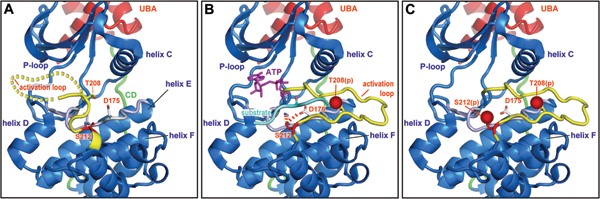
Model of conformational changes in the catalytic domain of MARK2. The model is based on the X-ray structure of MARK2 [PDB: 1Y8G] [24]. The catalytic loop (grey) is located deep in the cleft between the small and the large lobes of the kinase domain (blue). The ubiquitin-associated (UBA) domain (red) in the back is linked to the large lobe by a stretch that contains the common docking (CD) site (green). **(A) **In the inactive state, the catalytic loop (yellow; partly disordered and modeled here as dotted line) is folded back into the cleft and resides underneath the ATP-binding loop (P-loop). Both T208 and S212 point to the right and are accessible for kinases, for example, T208 to MARKK [17] and S212 to GSK3β [19]. **(B) **In the activated state, phosphorylation of the T208 (indicated by red sphere) results in a reorientation of the activation loop. It becomes folded out of the cleft and resides between helix C and helix F, stabilized by interactions of the pT208 to residues in helix C. S212 is now fixed by hydrogen bonds towards K177 and D175 in the catalytic loop (grey). The catalytic pocket opens up and allows entry of ATP (violet) and substrate (tau peptide, cyan), which aligns with the catalytic (grey) and the activation loop (yellow). **(C) **Phosphorylation of S212, or mutation to alanine or glutamate, disrupts the stabilizing interaction between the activation loop (yellow) and the catalytic loop (grey), resulting in an inactive kinase. Furthermore, it is likely that the phosphate of pS212 (indicated by a red sphere) will interfere with the correct alignment of the substrate within the catalytic cleft.

The T-loop is anchored to the base of the active site cleft via the Mg^2+ ^binding loop starting with the DFG motif (residues 193–195). In inactive MARK, the T-loop is partially disordered and assumes a variety of conformations. It is folded over the cleft in the inactive state (Figure 2A) so that the access of the substrate peptide and ATP are blocked. As with many other RD kinases, activation of MARK can be achieved by phosphorylating the T-loop (at T208). By analogy with the structure of other kinases in the active state, phosphorylation of this residue likely triggers reorientation and stabilization of the T-loop leading to an 'active' conformation that allows binding of the substrate molecule (Figure 3).

The catalytic domain has a carboxy-terminal extension that starts with a highly charged four residue motif (ExxE, x = E or D, except for MARK4, EGEE) that resembles the common docking (CD) site in MAP kinases [27] and may represent an attachment of upstream or downstream regulatory cofactors. This motif is followed by an extended chain of amino acid residues that ends with the UBA domain, a small, globular domain that binds close to the amino terminus of the catalytic domain. The generic UBA domain consists of three short helices (α1–3) folded in a characteristic helical bundle. In all MARK crystals that have been solved so far, including constructs from MARK isoforms 1, 2, and 3, the UBA domain is unusually folded: helix α3 is inverted compared to the typical fold (Figure 2B).

## Mechanisms of MARK regulation

**Figure 4:**
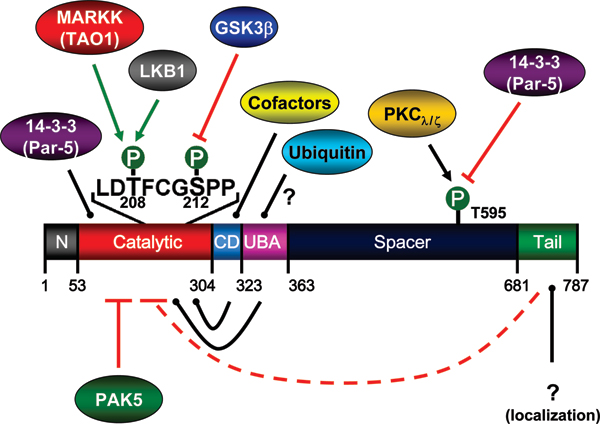
Modes of regulating MARK. The diagram summarizes known or plausible modes of MARK regulation that would affect the phosphorylation of tau and hence the stability of microtubules and the aggregation of tau. Activation via phosphorylation by MARKK (red) or LKB1 (grey) at T208 in the activation loop [17,18]. Inhibition by binding of PAK5 (green) to the catalytic domain [30]. Inhibition via phosphorylation by GSK3β (blue) at S212 in the activation loop [19]. Regulation by interaction of the UBA domain with ubiquitin (cyan) (not proven but suggested by X-ray structure) [24]. Regulation by interaction of the CD motif with a cofactor (yellow), in analogy with MAP kinases where upstream or downstream kinases can be bound [24,27]. Localization by interaction of the catalytic domain with the adaptor protein 14-3-3 (violet) (in analogy with *Drosophila *Par-1) [30,31]. This interaction does not depend on prior phosphorylation of MARK. Localization and probably inhibition by interaction of the spacer domain with 14-3-3 (violet), after prior phosphorylation by atypical protein kinase C (aPKC; orange), which creates a 14-3-3 binding motif on MARK [32,33]. Interaction between the carboxy-terminal tail and the amino-terminal header or catalytic domain (dotted line), creating a folded and inhibited MARK molecule (proposed for the yeast homolog Kin-1) [34].

As described above, MARK/Par-1 kinases can be activated by phosphorylation of a conserved threonine in the activation loop, T208 in MARK2 (Figure 4). Two upstream activating kinases have been identified, MARKK/TAO-1 [17] and LKB1 [18]. MARK2 purified from brain is, at least partially, also found to be phosphorylated at a second site, S212, adjacent to T208 [15]. This particular serine can be phosphorylated by GSK3β and, as a result, MARK becomes inactive. The inhibitory phosphorylation by GSK3β even overrides previous activation by MARKK or LKB1 at T208 [19]. These findings confirm earlier data, where site-directed mutagenesis of this residue to a phosphoserine-mimicking glutamate suggested that phosphorylation might be inhibitory [17]. X-ray analysis of the catalytic domains of MARK1 and MARK2 confirm the important function of this particular serine in stabilizing the activation loop [23,24]. This is illustrated in the structural model of MARK2 (Figure 3). In the inactive state of the kinase, the activation loop folds deep into the catalytic cleft, blocking the entry of the ATP and the substrate peptide (tau). Upon activation, the activation loop folds out of the catalytic cleft and resides between helix C and helix F, opening the cleft for ATP and the substrate. In this conformation, the S212 forms hydrogen bonds to the catalytic aspartate (D175) and a nearby lysine (K177), as proposed for PKA (PKA residues are T201, D166, and K168) [28]. This interaction, together with the contacts of the phosphorylated T208 to helix C, fixes the activation loop in the open conformation that is crucial for activity. Mutation of S212 to either alanine or glutamate disrupts this stabilizing interaction, and the same is achieved when S212 is phosphorylated. Since the activation loops of the four MARK isoforms are nearly identical in sequence, these results are likely to hold for all.

The activity of MARK is additionally regulated by several mechanisms that all lead to reduced activity (Figure 4) [29]. First, the activity is modulated by interaction with other proteins. Using a yeast two-hybrid screen, we identified the Ste20-kinase PAK5, which can bind to the catalytic domain of MARK, resulting in inhibition [30]. Furthermore, MARK can also interact with the scaffold protein 14-3-3/Par-5 (one of the conserved polarity genes). Two different modes have been proposed for this interaction: 14-3-3 can bind in a phosphorylation-independent manner to a fragment containing the catalytic domain and the linker to the UBA domain of MARK/Par-1 [31]. Alternatively, 14-3-3 can bind to the spacer domain after phosphorylation by atypical protein kinase C [32,33]. These interactions not only regulate MARK spatially by altering its localization, but also inhibit the catalytic activity of the enzyme, probably by stabilizing the inhibitory interaction of the tail domain with the amino-terminal header or the catalytic domain ([34], and our unpublished data). The structural analysis of MARK [24] suggests further regulatory interactions with yet unknown proteins and the UBA domain – for example, poly-ubiquitin – leading to intracellular signaling. Finally, the CD site is known in MAP kinases for multiple interactions with upstream and downstream effectors [27]; this motif can be found in all MARK isoforms [24].

## List of abbreviations used

AD: Alzheimer's disease; AMPK: adenosine monophosphate-activated protein kinase; BRSK1/2: brain-selective kinase 1/2; CD: common docking; GSK3β: glycogene synthase kinase 3β; MAP: microtubule-associated protein; MARK: MAP-microtubule affinity regulating kinase; MELK: maternal embryonic leucine zipper kinase; SNF1: sucrose non-fermenting 1; UBA: ubiquitin-associated.

## Competing interests

The authors declare that they have no competing interests.
